# In Vivo Antiviral Activity of Baloxavir against PA/I38T-Substituted Influenza A Viruses at Clinically Relevant Doses

**DOI:** 10.3390/v15051154

**Published:** 2023-05-11

**Authors:** Takayuki Kuroda, Keita Fukao, Shinpei Yoshida, Ryoko Oka, Kaoru Baba, Yoshinori Ando, Keiichi Taniguchi, Takeshi Noshi, Takao Shishido

**Affiliations:** 1 Shionogi & Co., Ltd., Osaka 561-0825, Japan; 2 Shionogi TechnoAdvance Research, Co., Ltd., Osaka 561-0825, Japan

**Keywords:** baloxavir, oseltamivir, influenza, PA/I38T, antiviral drugs

## Abstract

Although the prevalence of polymerase acidic (PA)/I38T strains of influenza virus with reduced susceptibility to baloxavir acid is low, there is a possibility of emergence under selective pressure. Furthermore, the virus may be transmitted between humans. We investigated the in vivo efficacy of baloxavir acid and oseltamivir phosphate against influenza A subtypes H1N1, H1N1pdm09, and H3N2, with PA/I38T substitution, at doses simulating human plasma concentrations. A pharmacokinetic/pharmacodynamic analysis was performed to strengthen the validity of the findings and the applicability in a clinical setting. Although the antiviral effect of baloxavir acid was attenuated in mice infected with PA/I38T-substituted viral strains compared with the wild type (WT), baloxavir acid significantly reduced virus titers at higher—but clinically relevant—doses. The virus titer reduction with baloxavir acid (30 mg/kg subcutaneous single dose) was comparable to that of oseltamivir phosphate (5 mg/kg orally twice daily) against H1N1 and H1N1pdm09 PA/I38T strains in mice, as well as the H3N2 PA/I38T strain in hamsters. Baloxavir acid demonstrated an antiviral effect against PA/I38T-substituted strains, at day 6, with no further viral rebound. In conclusion, baloxavir acid demonstrated dose-dependent antiviral effects comparable to that of oseltamivir phosphate, even though the degree of lung virus titer reduction was diminished in animal models infected with PA/I38T-substituted strains.

## 1. Introduction

Influenza is one of the most problematic acute respiratory infections worldwide, and along with its associated complications, it results in significant morbidity and mortality [1,2]. Antiviral drugs, including neuraminidase inhibitors (NAIs), adamantane derivatives, and a cap-dependent endonuclease (CEN) inhibitor, have been developed over the years for the treatment and prevention of influenza [2,3]. As most circulating influenza A viruses are resistant to the adamantane derivatives, rimantadine and amantadine, this group of drugs is no longer recommended [3]. NAIs, such as oseltamivir, zanamivir, and peramivir, are widely prescribed [2,3], as they are known to reduce the duration of influenza symptoms in otherwise healthy patients [4]. However, different mutant strains show reduced susceptibility to different NAIs. For example, the H1N1pdm09 NA/H275Y (N1 numbering; H274Y, N2 numbering) variant has decreased susceptibility to oseltamivir and peramivir but not to zanamivir [5]. Reduction in virus susceptibility to available antiviral drugs raises clinical concerns and the need to develop novel therapeutic strategies, such as CEN inhibitors.

Baloxavir marboxil is currently the only CEN inhibitor available for clinical use [6]. It is a prodrug that is hydrolyzed to its active form, baloxavir acid, following oral administration, and it acts by inhibiting the CEN residing in the polymerase acidic (PA) subunit of the influenza virus polymerase. In animal models and clinical trials, compared with placebo, a single dose of baloxavir marboxil has been shown to have a greater antiviral effect than NAIs [7,8,9,10,11,12]. In these studies, it shortened the duration of symptoms in both otherwise healthy patients, as well as those at higher risk of complications [7,8]. Baloxavir marboxil has demonstrated antiviral activity against influenza A, B, C, and D viruses [13]. However, influenza variants with PA mutations (such as E23K/G/R, A37T, I38T/M/F, and E199G), with reduced susceptibility to baloxavir marboxil, have been detected in treated patients [14,15,16,17]. The current World Health Organization Influenza Surveillance and Response System shows that the rate of these variants in circulating viruses is as low as < 0.1% globally [18,19,20,21]. The substitution of isoleucine for threonine, at position 38 of the PA subunit within the active site of CEN (PA/I38T), is a major pathway for reduced susceptibility to baloxavir marboxil [14]. Although no additional mutations that improve the fitness of the PA/I38T strains have been identified [22], there are sporadic reports of this mutant virus in children with no prior exposure to baloxavir acid [23]. This suggests the possibility of the mutant strain emerging under the selective pressure of baloxavir acid, as well as through human-to-human transmission [22,23]. Owing to the low frequency of clinical cases of PA/I38T baseline infections, the clinical efficacy of baloxavir marboxil and NAIs against these infections is difficult to establish, and the clinical implications remain unclear [24,25,26].

This study aimed to examine the antiviral effect of baloxavir acid and oseltamivir phosphate against influenza A viruses harboring the PA/I38T mutation in preclinical models. We used influenza A subtypes H1N1, H1N1pdm09, and H3N2 and their variants, with PA/I38T or NA/H275Y mutations. The in vitro antiviral activity of baloxavir acid was established using plaque inhibition assays, and the in vivo activity was investigated by measuring lung virus titers. Baloxavir acid was administered subcutaneously to maintain human-like plasma concentration. Doses that provide similar pharmacokinetic (PK) exposure in animal (mouse and hamster) models to that seen in human clinical studies were used. A pharmacokinetic/pharmacodynamic (PK/PD) analysis was conducted to determine the parameter that predicted the antiviral effect of baloxavir acid. This was used to facilitate translation into a clinical setting.

## 2. Materials and Methods

### 2.1. Compounds

Baloxavir acid was synthesized at Shionogi & Co., Ltd. (Osaka, Japan). Baloxavir suspension was prepared by suspending baloxavir acid in 0.5% (*w*/*v*) methylcellulose using an agate mortar and pestle. Oseltamivir phosphate was purchased from Sequoia Research Products Ltd. (Pangbourne, UK) and dissolved in 0.5% (*w*/*v*) methylcellulose. For the PK/PD studies, baloxavir acid was dissolved in 10% (*w*/*v*) Tween 80 and 0.5% (*w*/*v*) vinylpyrrolidone-vinyl acetate copolymer in sodium carbonate-sodium hydrogen carbonate buffer under heating, and the pH was adjusted to ~9. For dosing, each solution or suspension was diluted with the same respective vehicle.

### 2.2. Animals

All animal studies were conducted under applicable laws and guidelines and with the approval of the Shionogi Animal Care and Use Committee. The study is reported in accordance with ARRIVE guidelines.

Specific pathogen-free 6-week-old female BALB/c mice (Charles River Laboratories Japan, Inc., Yokohama, Japan) or Syrian hamsters (Japan SLC Inc., Shizuoka, Japan) were used.

#### 2.2.1. Sample Size

A total of 1441 mice and 84 hamsters were used in the study. The number of animals to be used was decided based on the results of preliminary tests while considering the reduction in the number of animals as much as possible. There were no excluded experimental units or data points.

#### 2.2.2. Inclusion and Exclusion Criteria

Animals with any abnormalities in initial body weight or general condition were not included. There were no other inclusion/exclusion criteria. As a result, of the 1541 mice and 84 hamsters prepared for the experiments, 1441 mice (≈94%) and all the hamsters were used.

#### 2.2.3. Animal Housing

All mice and hamsters were maintained in a temperature and relative humidity-controlled environment (20–26 °C and 30–70%, respectively), with a normal 12 h light and 12 h dark cycle. There were two hamsters housed per sterile cage, with chip paper bedding, paper towel, and cardboard tubes for environmental enrichment. Between 5 and 10 mice were housed per cage, with chip paper bedding and wooden bite stick for environmental enrichment. Standard chow diet (CE-2, CLEA Japan) and water were available ad libitum.

### 2.3. Cells and Viruses

Madin–Darby canine kidney (MDCK) cells were obtained from the European Collection of Authenticated Cell Cultures (Wiltshire, UK) and maintained using the method described previously [9].

The A/Osaka/129/2009 (H1N1pdm09) wildtype (WT) strain, named as H1N1pdm09.WT, was obtained from the Osaka Prefectural Institute of Public Health. The A/Osaka/129/2009-PA/I38T mutant strain, named as H1N1pdm09.PA/I38T, was prepared by passaging clinically isolated H1N1pdm09.WT in MDCK cells and subjecting them to drug pressure under baloxavir acid. The rgA/WSN/33 (H1N1) WT, PA/I38T, and NA/H275Y strains (rgH1N1.WT, rgH1N1.PA/I38T, and rgH1N1.NA/H275Y, respectively), as well as rgA/Victoria/3/75 (H3N2) WT and PA/I38T strains (rgH3N2.WT and rgH3N2.PA/I38T, respectively), were prepared at Shionogi & Co. Ltd. using reverse genetics, as described previously [14]. Virus stock was diluted with Minimum Essential Medium (Life Technologies, Inc., Grand Island, NY, USA) to obtain the appropriate titer of the virus suspension.

### 2.4. Plaque Reduction Assay

The susceptibility of H1N1pdm09.WT and H1N1pdm09.PA/I38T was determined using the plaque reduction assay described previously [14].

### 2.5. Anesthesia

Mice and hamsters were anesthetized upon virus inoculation by the intramuscular administration of an anesthetic solution containing medetomidine hydrochloride, midazolam, and butorphanol tartrate. For experiments analyzing plasma concentrations of baloxavir acid in non-infected animals, mice were anesthetized with isoflurane, and hamsters were unanesthetized upon blood sampling.

### 2.6. Drug Administration

When administered orally, baloxavir marboxil results in a shorter baloxavir acid plasma half-life in animal models than that observed in humans [11,27]. To maintain the comparable plasma concentrations, we therefore administered baloxavir acid suspension subcutaneously. We used clinically relevant doses of baloxavir acid in mice to mimic human exposure. For doses ≤ 10 mg/kg, one injection was administered subcutaneously to the “back of the neck”. In the case of 20 or 30 mg/kg doses, baloxavir acid was administered subcutaneously to two or three sites on the “back”, respectively. Oseltamivir phosphate was administered at a corresponding therapeutic dose of 5 mg/kg, orally, twice daily. The vehicle was an aqueous solution of 0.5% [*w*/*v*] methylcellulose that could be administered either subcutaneously or orally.

### 2.7. Antiviral (H1N1) Studies in Mice

#### 2.7.1. Examination of Virus Titers

Mice were euthanized by cervical dislocation under anesthesia, and virus titers in lung homogenates were determined 6 days post-infection (24 h after treatment). Lung homogenates were prepared by adding 2 mL of DPBS to approximately 200 mg of lungs. To measure lung virus titers, serial dilutions of lung homogenates were inoculated onto confluent MDCK cells, as described previously [9]. The presence of cytopathic effects was determined microscopically, and the virus titers were calculated as log_10_ median tissue culture infectious doses (TCID_50_)/mL. When no cytopathic effect was observed in the lowest dilution (10-fold), the titer of undetected virus was defined as 1.5 log_10_ TCID_50_/mL, except for the experiment assessing the time course of virus titers (Supplementary Figure S1). In this study, when no cytopathic effect was observed in the lowest dilution (5-fold), the titer of undetected virus was defined as 1.35 log_10_ TCID_50_/mL.

#### 2.7.2. Virus Growth Kinetics

Anesthetized mice were intranasally inoculated with 300 TCID_50_ of rgH1N1.WT, rgH1N1.PA/I38T, rgH1N1.NA/H275Y, H1N1pdm09.WT, or H1N1pdm09.PA/I38T; 100 TCID_50_ or 300 TCID_50_ of rgH1N1.PA/I38T; 10,000 TCID_50_ of rgH1N1.WT, rgH1N1.PA/I38T, or rgH1N1.NA/H275Y. The lung virus titers of 5–10 mice in each group were measured on days 1–7 post-infection in the group infected with 300 TCID_50_ rgA/WSN/33 strains and on days 3–7 post-infection in those infected with 300 TCID_50_ H1N1pdm09 strains and 10,000 TCID_50_ H1N1 strains.

#### 2.7.3. Dose-Dependency of Virus Inhibition after Treatment with Baloxavir Acid in Mice Infected with rgH1N1.WT or rgH1N1.PA/I38T

Anesthetized mice were intranasally inoculated with 300 TCID_50_ of rgH1N1.WT or rgH1N1.PA/I38T virus suspension, and then, they were treated with the respective antiviral 5 days post-infection. Mice infected with rgH1N1.WT were treated with baloxavir acid (0.03, 0.1, 0.3, 1, or 3 mg/kg single doses; n = 10/group), oseltamivir phosphate (n = 10/group), or vehicle (10 mL/kg subcutaneous single dose; n = 10/group). Mice infected with rgH1N1.PA/I38T were treated with baloxavir acid (1, 3, 10, or 30 mg/kg single dose or 30 mg/kg four times daily [every 3 h]; n = 10/group), oseltamivir phosphate (n = 10/group), or vehicle (10 mL/kg subcutaneous single dose; n = 10/group).

#### 2.7.4. Antiviral Effect of Baloxavir Acid at Doses Simulating Human Plasma Concentrations in Mice Infected with rgH1N1.WT, rgH1N1.PA/I38T, or rgH1N1.NA/H275Y

Anesthetized mice were intranasally inoculated with 300 TCID_50_ of rgH1N1.WT, rgH1N1.PA/I38T, or rgH1N1.NA/H275Y. They were then treated with baloxavir acid (10 or 30 mg/kg single dose; n = 20/group), oseltamivir phosphate (n = 20/group), or a vehicle (10 mL/kg subcutaneous single dose; n = 19–20/group) 5 days post-infection.

#### 2.7.5. Antiviral Effect of Baloxavir Acid at Doses Simulating Human Plasma Concentrations in Mice Infected with H1N1pdm09.WT or H1N1pdm09.PA/I38T

Anesthetized mice were intranasally inoculated with 300 TCID_50_ of H1N1pdm09.WT or H1N1pdm09.PA/I38T virus suspension. They were then treated with baloxavir acid (10 or 30 mg/kg subcutaneous single dose; n = 10/group), oseltamivir phosphate (n = 10/group), or vehicle (10 mL/kg subcutaneous single dose; n = 10/group) 5 days post-infection.

#### 2.7.6. Time Course of Lung Virus Titers in H1N1pdm09.PA/I38T Infections

Anesthetized mice were intranasally inoculated with 300 TCID_50_ of H1N1pdm09.PA/I38T virus suspension. They were then treated with baloxavir acid (10 or 30 mg/kg subcutaneous single dose; n = 5–8/group), oseltamivir phosphate (for 3 days; n = 5–8/group), or vehicle (10 mL/kg subcutaneous single dose; n = 5–8/group) 5 days post-infection. The virus titers in lung homogenates were determined on days 5, 6, 7, and 8 post-infection.

#### 2.7.7. Time Course of Lung Virus Titers in rgH1N1.PA/I38T Infections

Anesthetized mice were intranasally inoculated with 300 TCID_50_ of rgH1N1.PA/I38T and treated with baloxavir acid (10 or 30 mg/kg single dose; n = 5/group) or vehicle (10 mL/kg subcutaneous single dose; n = 5/group) 5 days post-infection. The virus titers in lung homogenates were determined on days 6, 7, 8, 11, and 14 post-infection.

#### 2.7.8. Sequence Analysis of PA Genes

Viral RNA was isolated from lung homogenates of infected mice using the Qiagen^®^ One-Step RT-PCR kit (Qiagen, Hilden, Germany). The PA region of the influenza virus was amplified by nested reverse transcription polymerase chain reaction. The primer sequences used are listed (Supplementary Table S1). Sanger sequence analysis of the PA region was performed by Eurofins Genomics (Tokyo, Japan).

#### 2.7.9. Effect on Body Weight in Mice

Anesthetized mice were intranasally inoculated with 10,000 TCID_50_ of rgH1N1.WT, rgH1N1.PA/I38T, or rgH1N1.NA/H275Y. The initial body weights of all the mice were recorded on the day of infection. Mice were assigned to each group one day prior to or on the first day of treatment. Assignment was based upon the uniformity of mean body weight and the mean proportion of body weight to initial body weight among groups. From each group, 10–20 infected mice were treated with baloxavir acid (10 or 30 mg/kg subcutaneous single dose and vehicle, 10 mL/kg orally, twice daily, for 5 days), oseltamivir phosphate (5 mg/kg orally, twice daily, for 5 days and vehicle 10 mL/kg subcutaneous single dose), or vehicle (10 mL/kg subcutaneous single dose and orally, twice daily, for 5 days). For 10 days and 14 days after infection, mice were examined daily for body weight and survival. Mice were regarded as dead if their body weights were lower than 70% of the initial body weight according to the humane endpoint. These mice were euthanized. In the analysis of the evaluation of the effect on body weight change, mice who died or reached the endpoint were extrapolated as 70% of the body weight on the day of infection.

### 2.8. Antiviral (H3N2) Studies in Hamsters

Anesthetized hamsters were intranasally inoculated with 300 TCID_50_ of rgH3N2.WT or rgH3N2.PA/I38T virus suspension. At 1 day post-infection, infected hamsters were treated with baloxavir acid (10 or 30 mg/kg subcutaneous single dose; n = 8/group), oseltamivir phosphate (5 mg/kg orally twice daily; n = 8/group), or vehicle (5 mL/kg subcutaneous single dose; n = 8/group). Hamsters were euthanized by exsanguination, and the lung tissues were removed. The lung homogenates were prepared by adding 6 mL of DPBS to approximately 600 mg of lungs. Virus titers in lung homogenates were determined 2 days post-infection (24 h after treatment).

### 2.9. PK/PD Analysis

#### 2.9.1. PK Studies in Non-Infected Mice

To determine whether blood exposure of baloxavir acid could be increased linearly by injecting the drug into multiple sites, baloxavir acid (prepared using baloxavir acid in suspension: 1 mg/mL for mice; 5 mg/mL for hamsters) was injected subcutaneously at different sites in mice (n = 3/group) and hamsters (n = 3/group).

For mice, ≈10 mg/kg (0.2 mg/mouse) of the suspension was administered to one (≈10 mg/kg), two (≈20 mg/kg), or three (≈30 mg/kg) sites. Blood (≈0.025–0.4 mL/time point) was taken from the inferior vena cava, heart, or tail vein at 2, 4, 8, 24, 48, 120, and 216 h (two and three sites) or 2, 4, 8, 24, 48, 120, 168, and 216 h (one site) after dosing. The maximum plasma concentration (C_max_) and area under the curve, from 0 to the time of the last quantifiable concentration (AUC_last_), were calculated by WinNonlin (Certara USA Inc., Princeton, NJ, USA) based on a non-compartment model with uniform weighting. In hamsters, 10 mg/2 mL/kg of the suspension was administered to one (10 mg/kg) or three (total of 30 mg/kg) sites. Blood (≈0.1 mL/time point) was collected from the jugular vein at 1, 4, 8, 24, 48, and 72 h after dosing. In both mice and hamsters, the plasma concentration was determined using liquid chromatography with tandem mass spectrometry (LC-MS/MS). We found that administering 1, 2, and 3 subcutaneous injections (≈10, 20, and 30 m/kg) to separate sites on the back of the mice resulted in a linear increase in baloxavir acid at 24 h (C_24_) levels (26.1 ± 1.3, 47.4 ± 4.9, and 70.8 ± 13.9 ng/mL, respectively, n = 3; Supplementary Figure S2). Similarly, administering one and three subcutaneous injections (≈10 and 30 mg/kg) to separate sites on the back of the hamsters resulted in a linear increase in C_24_ levels (17.3 ± 3.3 and 45.7 ± 19.7 ng/mL, respectively, n = 3; Supplementary Figure S2). To achieve optimal plasma concentrations with a dose of 30 mg/kg, we therefore administered three subcutaneous injections of 10 mg/kg to separate sites in this study.

To compare the PK parameters of baloxavir acid in humans, mice, and hamsters, the human oral PK data were derived from a study on population PK parameters and exposure–response analyses in adults and adolescents [26]. All mice (n = 3) and hamsters (n = 3) received one (10 mg/kg) subcutaneous suspension of baloxavir acid (1 mg/mL for mice; 2 mg/mL for hamsters). Plasma concentrations of baloxavir acid were determined by LC-MS/MS, using blood (mice: ≈0.025–0.4 mL/time point; hamster: ≈0.1 mL/time point) taken from the inferior vena cava or heart of anesthetized mice (at 2, 4, 8, 24, 48, 120, 168, and 216 h after dosing) or from the jugular vein of unanesthetized hamsters (at 1, 4, 8, 24, 48, and 72 h after dosing). Assuming dose linearity, plasma concentrations of baloxavir acid for a 30 mg/kg suspension in mice and hamsters were estimated from the measured plasma concentration data for the 10 mg/kg suspension (Supplementary Figure S3).

#### 2.9.2. PK/PD Studies in Mice Infected with rgH1N1.PA/I38T

For the PD study, mice infected with rgH1N1.PA/I38T were treated with baloxavir acid (2, 4, 8, 16, 32, or 64 mg/kg subcutaneous single dose; 0.5, 1, 2, 4, 8, or 16 mg/kg, subcutaneously twice daily; 0.125, 0.25, 0.5, 1, 2, or 4 mg/kg subcutaneously four times daily [n = 8/group]), oseltamivir phosphate (5 mg/kg orally twice daily; n = 10/group), or vehicle (10% [*w*/*v*] Tween 80 and 0.5% [*w*/*v*] vinylpyrrolidone-vinyl acetate copolymer in sodium carbonate-sodium hydrogen carbonate buffer, subcutaneously, in a single dose; n = 10/group) for 1 day. After 24 h, mice were euthanized by cervical dislocation, and lung virus titers were evaluated.

To obtain the PK data, mice infected with rgH1N1.PA/I38T were treated with baloxavir acid (0.25, 1, 4, 16, or 64 mg/kg, subcutaneous single dose) and blood was sequentially taken from the tail vein at 0.25, 0.5, 1, 2, 4, 6, 8, or 24 h after dosing (n = 3/group). Plasma concentrations of baloxavir acid were determined by LC-MS/MS and averaged by dose and nominal time. The mean plasma concentrations, at each sampling time and dose level, were used for the PK analysis. The C_max_, plasma concentration at the end of the dosing interval after the first dosing (C_τ_), and area under the curve, from time 0 to 24 h after dosing (AUC_0–24_), were calculated by WinNonlin based on a non-compartment model with uniform weighting. The same PK parameters for the doses that were not administered to infected mice for PK evaluation (0.125, 0.5, 2, 8, and 32 mg/kg) were mathematically scaled by extrapolation or interpolation, based on the PK parameters in infected mice dosed at 0.25, 1, 4, 16, or 64 mg/kg, using the method described previously [11]. Each parameter’s value at the 0.125 mg/kg dose was calculated as 1/2 × the parameter’s value at the 0.25 mg/kg dose; the parameters for the remaining values (0.5, 2, 8, and 32 mg/kg) were calculated as 2/3 × the value at the nearest lower dose + 1/3 × the value at the nearest higher dose, as described previously^11^ (Supplementary Table S2).

The linear model (*y* = E_0_–β*x*) was used to investigate the relationship between antiviral activity and PK parameters, following subcutaneous baloxavir acid dosing, in the rgH1N1.PA/I38T infection model. This model was applied to virus titer data, derived from individual mice in the PD studies, and to the PK parameters that were calculated from the observed mean plasma concentrations at each dose and time point (for doses 0.25, 1, 4, 16, or 64 mg/kg), as well as the PK parameters for 0.125, 0.5, 2, 8, and 32 mg/kg doses that were mathematically estimated by extrapolation or interpolation based on the PK parameters at 0.25, 1, 4, 16, or 64 mg/kg. E_0_ is the baseline effect, and β is a regression coefficient. In this case, AUC_0–24_, C_max_, C_24_, and C_τ_ were used as the logarithmic values. Model fitness was evaluated by the coefficient of determination, *R^2^*, adjusted for degrees of freedom (Supplementary Table S2).

### 2.10. Statistical Analyses

Virus titers and body weights on each day, after treatment with baloxavir acid, were compared with the respective control groups (oseltamivir phosphate and vehicle) and analyzed using the Dunnett’s multiple comparison method. Statistical analyses were performed using SAS Studio version 9.4 for Windows (SAS Institute, Cary, NC, USA). The two sided-adjusted *p*-values below 0.05 were considered statistically significant.

In the statistical analysis, it was assumed that the data showed a normal distribution. The normal distribution of the data was confirmed in the histogram, and we judged that the data met the assumption.

## 3. Results

### 3.1. In Vitro Drug Susceptibility of Influenza a Viruses to Baloxavir Acid and Oseltamivir Acid

The in vitro drug susceptibility of H1N1pdm09 WT and PA/I38T strains to baloxavir acid was tested using a plaque reduction assay. The drug sensitivity of H1N1pdm09.PA/I38T to baloxavir acid (measured as the half-maximal effective concentration [EC_50_]) was reduced by 25.74-fold (Table 1). Data from a previous study [14] showed that the presence of PA/I38T mutation in H1N1 (rgA/WSN/33) and H3N2 (rgA/Victoria/3/75) strains decreased the drug sensitivity to baloxavir acid by 27.24-fold and 56.59-fold, respectively. The drug sensitivity of rgH1N1.PA/I38T and rgH3N2.PA/I38T to oseltamivir acid (measured as the half-maximal inhibitory concentration [IC_50_] was comparable to respective WT strains (1.00-fold and 1.21-fold, respectively). The presence of NA/H275Y mutation in the H1N1 (rgA/WSN/33) strain decreased the drug sensitivity to oseltamivir acid by 226.12-fold, but it did not affect the drug sensitivity to baloxavir acid (0.77-fold change). Data for the drug sensitivity of the H1N1pdm09 (A/Osaka/129/2009) to oseltamivir acid were not available (Table 1).

### 3.2. Virus Growth Kinetics in a Non-Lethal Mouse Model

In a previous study [11], we established a non-lethal mouse model, using TCID_50_ of 100 with the natural strains, to evaluate the rapid reduction in virus burden following baloxavir marboxil treatment. Treatment was initiated 5 days post-infection followed by lung virus titer quantification at 6 days post-infection (24 h after treatment) [11]. However, the reverse genetic (rg) H1N1 strains in this experiment demonstrated weak viral growth at 100 TCID_50_ (Supplementary Figure S4). We therefore established a similar model using 300 TCID_50_ with the H1N1 (rgA/WSN/33) and H1N1pdm09 (A/Osaka/129/2009) strains. The viral growth kinetics for each virus tested (H1N1 and H1N1pdm09), from 3 to 6 days post-infection, were similar across the different strains, with minimal impact on body weight (Supplementary Figures S5 and S6).

### 3.3. PK Studies in Non-Infected Mice

To increase the validity of results in humans, we used baloxavir acid and oseltamivir phosphate at doses that achieved plasma concentrations similar to those recorded in humans. We compared human oral PK data derived from a previous study (on population PK parameters and exposure–response analyses in adults and adolescents) [26] to plasma concentrations of baloxavir acid in the animal models. The C_24_ in mice following a 10 mg/kg single dose was 26.1 ng/mL, and that of a simulated dose of 30 mg/kg was 78.3 ng/mL. These concentrations were within the 10th–90th percentile of the human C_24_ (Supplementary Figure S3). For oseltamivir phosphate, we used a corresponding therapeutic dose of 5 mg/kg, twice daily in mice, which has been shown to achieve the same AUC as an oral dose of 75 mg, twice daily, in humans in a previous study [28].

### 3.4. Dose-Dependent Antiviral Effect of Baloxavir Acid in Mice Infected with H1N1 WT and PA/I38T Strains

Mice were infected with rgH1N1.WT or rgH1N1.PA/I38T (300 TCID_50_) and treated with baloxavir acid, oseltamivir phosphate, or vehicle 5 days post-infection. Lung virus titers were evaluated 6 days post-infection (24 h after treatment).

**Figure 1:**
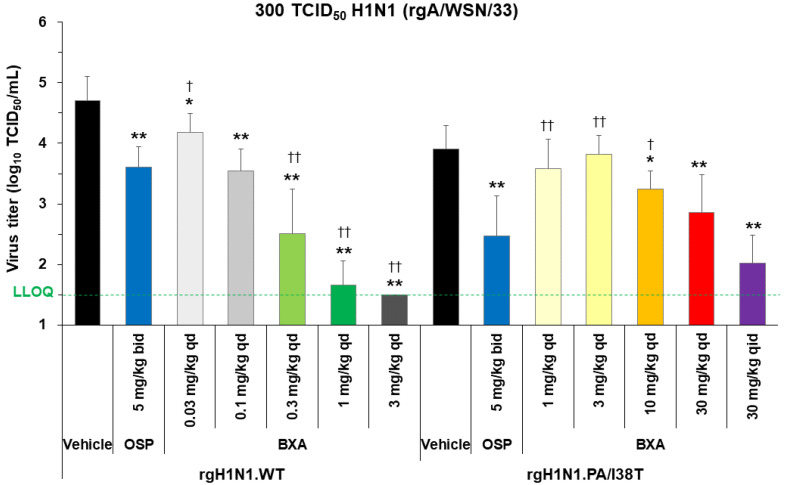
Dose dependency of virus inhibition after treatment with baloxavir acid, oseltamivir phosphate, or vehicle in mice infected with H1N1 WT or PA/I38T strains. Mice infected with 300 TCID_50_ rgH1N1.WT were treated with baloxavir acid (0.03–3 mg/kg qd), oseltamivir phosphate (5 mg/kg bid), or vehicle 5 days post-infection. Mice infected with 300 TCID_50_ rgH1N1.PA/I38T were treated with baloxavir acid (1–30 mg/kg qd or 30 mg/kg qid), oseltamivir phosphate (5 mg/kg bid), or vehicle 5 days post-infection. Lung virus titers were measured 6 days post-infection (24 h after the first antiviral dosing). The green dotted line shows the LLOQ. Each bar represents the mean ± standard deviation of 10 mice. * *p* < 0.05 and ** *p* < 0.0001 vs. vehicle, ^†^ *p* < 0.01 and ^††^ *p* < 0.0001 vs. oseltamivir phosphate (Dunnett’s test) bid, twice daily; BXA, baloxavir acid; LLOQ, lower limit of quantification; OSP, oseltamivir phosphate; qd, once daily; qid, four times daily; TCID_50_, median tissue culture infectious dose; WT, wild type.

Baloxavir acid showed a dose-dependent decrease in lung virus titers in both strains (Figure 1). In mice infected with the rgH1N1.WT, the virus titers in baloxavir acid-treated groups were significantly lower than vehicle-treated groups at all doses (*p* < 0.05). A single dose of baloxavir acid at 0.1 mg/kg showed a similar antiviral effect to that of oseltamivir phosphate (5 mg/kg, twice daily). The antiviral activity of baloxavir acid was significantly greater than oseltamivir phosphate at doses of 0.3 mg/kg and above (*p* < 0.0001). In rgH1N1.PA/I38T-infected mice, baloxavir acid demonstrated a significant decrease in virus titers, with doses of 10 mg/kg or more, compared with vehicle (*p* < 0.05) and a similar antiviral effect to oseltamivir phosphate at the 30 mg/kg dose, given once daily (*p* = 0.2925). To confirm dose dependency in this model, we used a high dose of 120 mg/kg/day (30 mg/kg, four times daily for 1 day, over a clinically equivalent dose). Notably, in PA/I38T-infected mice, this dose (120 mg/kg/day) reduced virus titers by 1.88 log_10_ TCID_50_/mL compared with vehicle (Figure 1). There was an overall shift in the amount of baloxavir acid needed to achieve the same level of inhibition for the PA/I38T strain as the WT strain. However, the doses of 10 and 30 mg/kg (single dose) still resulted in concentrations that were within the human C_24_ range (Supplementary Figure S3). These doses were, therefore, used in further experiments examining the antiviral effect of baloxavir acid.

### 3.5. Effect of Baloxavir Acid and Oseltamivir Phosphate on Lung Virus Titers in Mice Infected with H1N1 and H1N1Pdm09 WT and PA/I38T Strains

**Figure 2:**
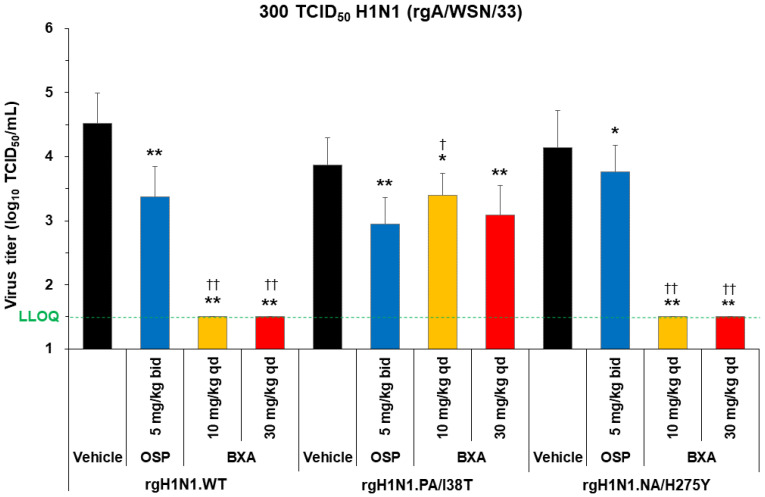
Antiviral effect of baloxavir acid at doses simulating human plasma concentrations in mice infected with H1N1 WT, PA/I38T, or NA/H275Y strains. Mice infected with 300 TCID_50_ rgH1N1.WT, rgH1N1.PA/I38T or rgH1N1.NA/H275Y were treated with baloxavir acid (10 or 30 mg/kg qd), oseltamivir phosphate (5 mg/kg bid), or vehicle 5 days post-infection. Lung virus titers were measured 6 days post-infection (24 h after the first antiviral dosing). The green dotted line shows the LLOQ. Each bar represents the mean ± standard deviation of 19–20 mice. * *p* < 0.01 and ** *p* < 0.0001 vs. vehicle, ^†^ *p* < 0.05 and ^††^ *p* < 0.0001 vs. oseltamivir phosphate (Dunnett’s test) bid, twice daily; BXA, baloxavir acid; LLOQ, lower limit of quantification; OSP, oseltamivir phosphate; qd, once daily; TCID_50_, median tissue culture infectious dose; WT, wild type.

**Figure 3:**
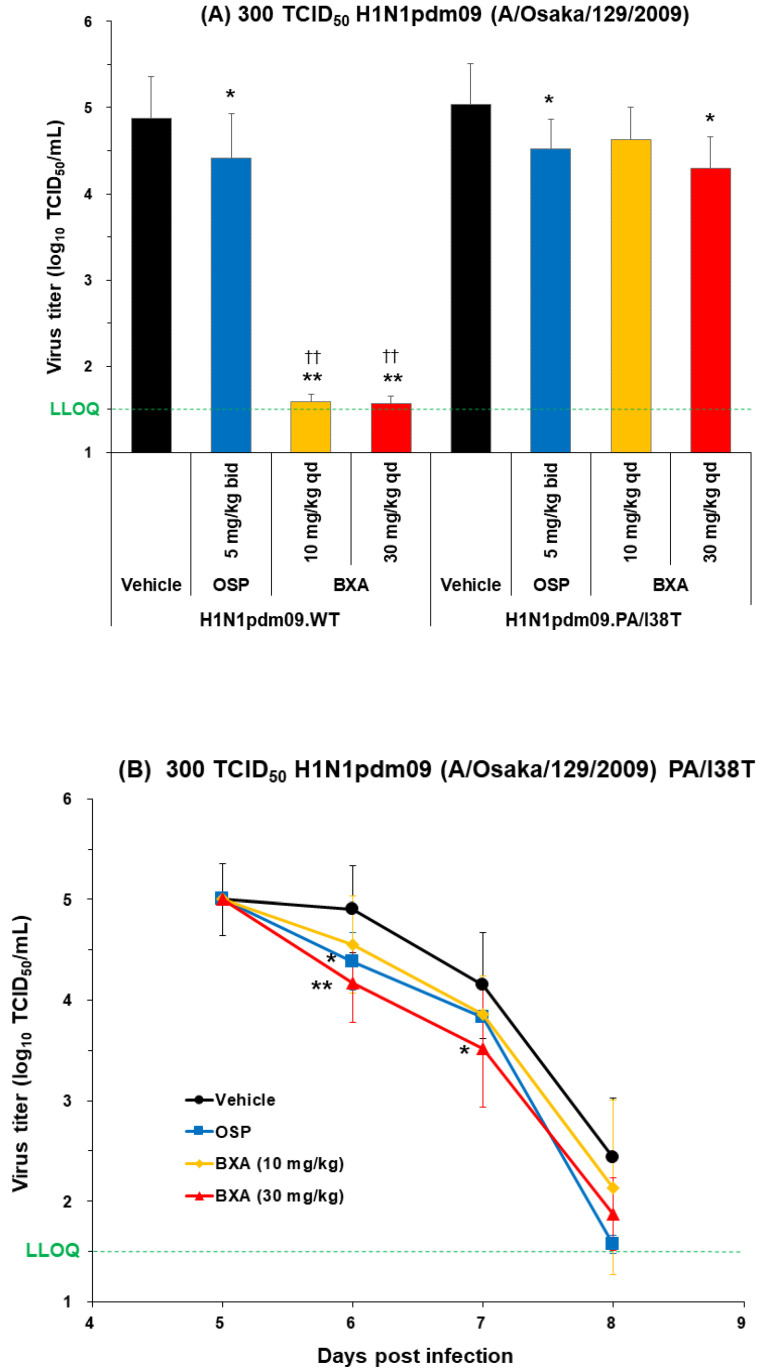
Antiviral effect of baloxavir acid at doses simulating human plasma concentrations in mice infected with H1N1pdm09 WT or PA/I38T strains. (**A**) Mice infected with 300 TCID_50_ H1N1pdm09.WT or H1N1pdm09.PA/I38T were treated with baloxavir acid (10 or 30 mg/kg qd), oseltamivir phosphate (5 mg/kg bid), or vehicle 5 days post-infection. Lung virus titers were measured 6 days post-infection in both strains. Each bar represents the mean ± standard deviation of 10 mice. * *p* < 0.05 and ** *p* < 0.0001 vs. vehicle, ^††^ *p* < 0.0001 vs. oseltamivir phosphate (Dunnett’s test). (**B**) Mice infected with 300 TCID_50_ H1N1pdm09.PA/I38T were treated with baloxavir acid (10 or 30 mg/kg qd for 1 day), oseltamivir phosphate (5 mg/kg bid for 3 days), or vehicle (qd for 1 day). Lung virus titers were measured on days 5, 6, 7, and 8 post-infection. The black lines with circles represent vehicle, the blue lines with squares represent oseltamivir phosphate, the yellow lines with diamonds represent baloxavir acid (10 mg/kg), and the red lines with triangles represent baloxavir acid (30 mg/kg). Data represent the mean ± standard deviation of 5–8 mice. * *p* < 0.05 and ** *p* < 0.01 vs. vehicle (Dunnett’s test). The green dotted line shows the LLOQ. bid, twice daily; BXA, baloxavir acid; LLOQ, lower limit of quantification; OSP, oseltamivir phosphate; qd, once daily; TCID_50_, median tissue culture infectious dose; WT, wild type.

Mice were inoculated with 300 TCID_50_ of rgH1N1.WT, rgH1N1.PA/I38T, rgH1N1.NA/H275Y, H1N1pdm09.WT, or H1N1pdm09.PA/I38T. Baloxavir acid (10 or 30 mg/kg; single dose) or oseltamivir phosphate (5 mg/kg, twice daily) was administered 5 days post-infection, and the lung virus titer was evaluated 6 days post-infection (Figure 2 and Figure 3A).

Baloxavir acid resulted in a significantly large drop in virus titers to below the lower limit of quantification (LLOQ; 1.5 log_10_ TCID_50_/mL) in mice infected with the rgH1N1.WT and rgH1N1.NA/H275Y, as well as almost to the LLOQ in mice infected with the H1N1pdm09.WT. The antiviral effect of baloxavir acid was diminished in mice infected with the PA/I38T strains, but the virus titers remained significantly lower than vehicle at all doses except with the 10 mg/kg dose against the H1N1pdm09.PA/I38T.

Oseltamivir phosphate demonstrated numerically different virus titer reduction across the different strains, but the virus titers were statistically lower compared with vehicle against all strains. The degree of virus titer reduction in oseltamivir phosphate was numerically smaller in the rgH1N1.NA/H275Y (0.38 log_10_TCID_50_/mL) than in the WT strain (1.15 log_10_TCID_50_/mL), suggesting reduced susceptibility to oseltamivir phosphate by NA/H275Y substitution.

Importantly, the reduction in lung virus titers with baloxavir acid (30 mg/kg, single dose) and oseltamivir phosphate (5 mg/kg, twice daily) was statistically similar for rgH1N1.PA/I38T and H1N1pdm09.PA/I38T (*p* = 0.4233 and *p* = 0.3078, respectively; Figure 2 and Figure 3A). Similar results were noted in the dose-dependency experiment for rgH1N1.PA/I38T (*p* = 0.2925; Figure 1). In the case of the NA/H275Y mutant variant (rgH1N1.NA/H275Y), baloxavir acid showed a significantly higher antiviral activity compared with oseltamivir phosphate (*p* < 0.0001; Figure 2).

### 3.6. Virus Titer Kinetics after Dosing

To determine whether mice infected with 300 TCID_50_ H1N1pdm09.PA/I38T and rgH1N1.PA/I38T remain virus free post-baloxavir acid treatment, virus titers were evaluated up to 8 and 14 days post-infection, respectively (Figure 3B and Supplementary Figure S1).

We did not observe any increase in virus titers during this time window (Figure 3B and Supplementary Figure S1). Moreover, the overall virus titers in H1N1pdm09.PA/I38T-infected mice continued to decrease over time; they were significantly lower with baloxavir than the vehicle-treated mice, on days 6 and 7 post-infection, at the dose of 30 mg/kg, and they were reduced to near the LLOQ on day 8 (1.87 log_10_TCID_50_/mL).

To monitor the emergence of further substitutions in addition to PA/I38T, during or after treatment with baloxavir acid, we conducted a Sanger sequence analysis of PA genes. We did not find any genotype changes, by observation, up to 14 days post-infection (9 days after baloxavir administration) in mice infected with the rgH1N1.PA/I38T and 8 days post-infection (3 days after baloxavir administration) in mice infected with the H1N1pdm09.PA/I38T.

### 3.7. Antiviral Effect of Baloxavir Acid in H3N2-Infected Hamsters

As mice are not susceptible to seasonal H3N2 viruses, we examined the effect of baloxavir acid on rgH3N2.WT and rgH3N2.PA/I38T using hamster models, in which H3N2 more efficiently proliferates [17].

**Figure 4:**
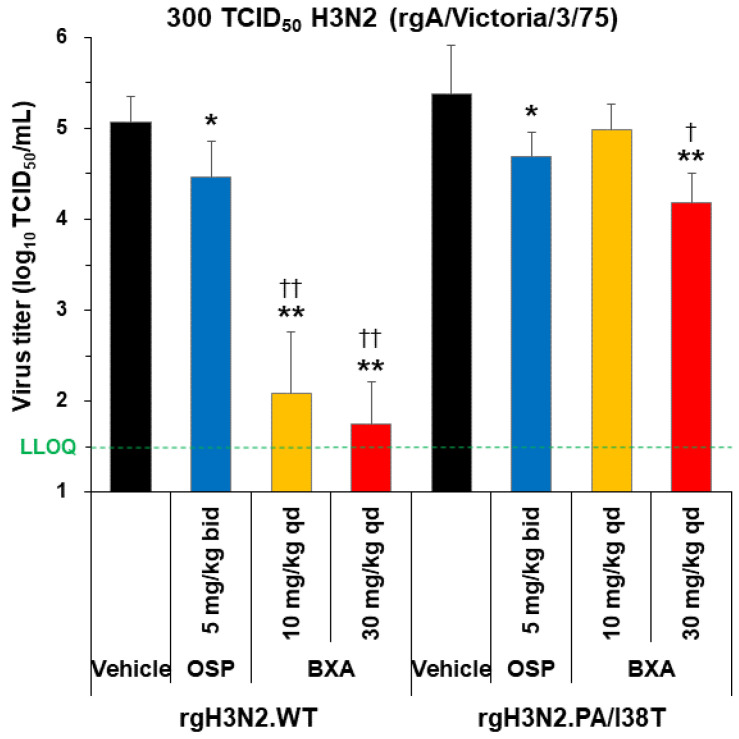
Antiviral effect of baloxavir acid at doses simulating human plasma concentrations in hamsters infected with H3N2 WT or PA/I38T strains. Hamsters infected with 300 TCID_50_ rgH3N2.WT or rgH3N2.PA/I38T were treated with baloxavir acid (10 or 30 mg/kg qd), oseltamivir phosphate (5 mg/kg bid), or vehicle 1 day post-infection. Lung virus titers were measured 2 days post-infection (24 h after the first antiviral dosing). The green dotted line shows the LLOQ. Each bar represents the mean ± standard deviation of 8 hamsters. * *p* < 0.05 and ** *p* < 0.0001 vs. vehicle, ^†^ *p* < 0.05 and ^††^ *p* < 0.0001 vs. oseltamivir phosphate (Dunnett’s test) bid, twice daily; BXA, baloxavir acid; LLOQ, lower limit of quantification; OSP, oseltamivir phosphate; qd, once daily; TCID_50_, median tissue culture infectious dose; WT, wild type.

In rgH3N2.WT-infected hamsters, baloxavir acid showed a significantly higher reduction in virus titers (at both 10 and 30 mg/kg single doses) than vehicle and oseltamivir phosphate (both *p* < 0.0001; Figure 4). Although the PA/I38T mutation significantly lowered the drug susceptibility towards baloxavir acid, a single dose of 30 mg/kg resulted in a statistically significant reduction in virus titers compared with the vehicle (*p* < 0.0001) and oseltamivir phosphate (*p* < 0.05; Figure 4).

### 3.8. PK/PD Analysis in Infected Mice

We performed a PK/PD analysis in a non-lethal mouse model infected with the rgH1N1.PA/I38T and treated it with baloxavir acid suspension.

PK parameters for the 0.25, 1, 4, 16, or 64 mg/kg doses of baloxavir acid in mice infected with rgH1N1.PA/I38T were calculated from the observed mean plasma concentration. Those for the 0.125, 0.5, 2, 8, and 32 mg/kg doses were mathematically scaled, assuming a linear relationship between the dose and PK parameters. These were correlated with the PD data on antiviral activity of baloxavir acid, determined from the virus titer data, using the linear model. Model fitness was evaluated by the coefficient of determination *R*^2^ adjusted for degrees of freedom. The adjusted *R*^2^ values were 0.633, 0.606, 0.627, and 0.672 for AUC_0–24_, C_max_, C_24_, and C_τ_, respectively. The C_τ_ was the parameter that demonstrated the highest *R*^2^, and it most closely correlated with lung virus titer 24 h after administration. A previous study conducted in a similar way, in mice infected with A/WSN/33 (H1N1) and treated with baloxavir acid, had also shown that the C_τ_ was the best PK parameter to predict virus titers [11] (Supplementary Table S3).

**Figure 5:**
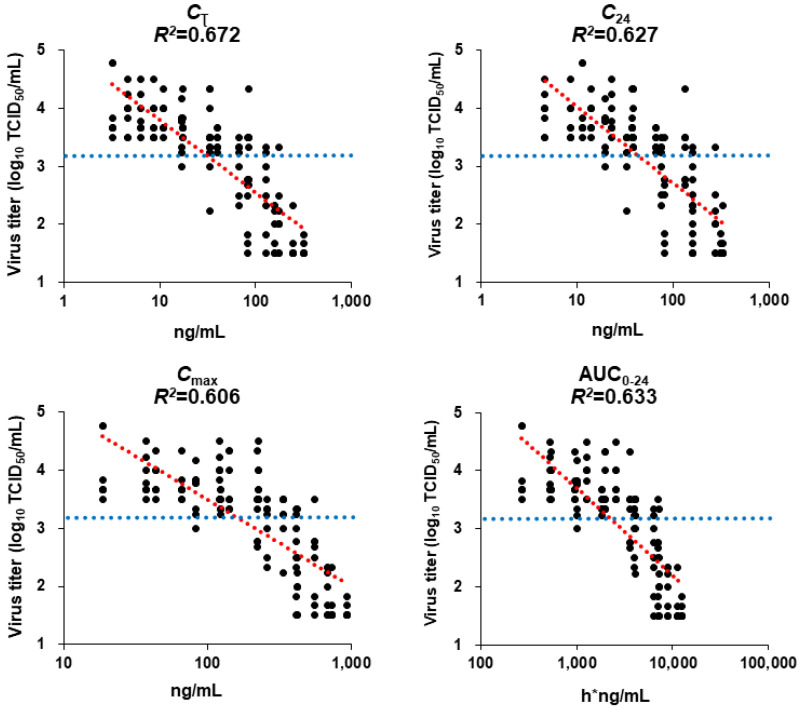
Estimated linear curves showing the relationship between pharmacokinetic parameters and lung virus titers in mice infected with H1N1 PA/I38T strain. Mice infected with rgH1N1.PA/I38T were treated with baloxavir acid (2, 4, 8, 16, 32, or 64 mg/kg qd; 0.5, 1, 2, 4, 8, or 16 mg/kg, bid; 0.125, 0.25, 0.5, 1, 2, or 4 mg/kg qid), oseltamivir phosphate (5 mg/kg bid), or vehicle for 1 day. Lung virus titers were measured, and the linear model was applied to evaluate the relationship between the pharmacokinetic parameters and virus titers. The blue dotted line shows the mean virus titers recorded after treatment with oseltamivir phosphate (3.17 log_10_ TCID_50_/mL). The red dotted line is the line of best fit. AUC_0–24_, the area under the curve from 0 to 24 h; bid, twice daily; C_τ_, plasma concentration at the end of the dosing interval after the first dosing; C_24_, plasma concentration at 24 h after the first dosing; C_max_, maximum plasma concentration; qd, once daily; qid, four times daily.

The mean virus titer achieved with the in vivo administration of oseltamivir phosphate was 3.17 log_10_ TCID_50_/mL in this model. The corresponding C_τ_ value of baloxavir acid that achieved a virus titer of 3.17 log_10_ TCID_50_/mL in rgH1N1.PA/I38T-infected mice was 31.6 ng/mL (Figure 5).

### 3.9. Inhibitory Effect of Baloxavir Acid on Weight Loss Due to Viral Infection in Murine Model

The sub-lethal infectious dose of 300 TCID_50_ did not cause any loss in body weight in rgH1N1.PA/I38T-infected mice. A dose to examine the inhibitory effect of baloxavir acid on weight loss due to viral infection was established by testing higher inoculation titers. The 10,000 TCID_50_ infectious dose resulted in 12.7% loss of body weight—but not lethality—at day 6 post-infection in the PA/I38T strain, and it was considered an appropriate dose for this experiment (Supplementary Figure S6). Viral growth and loss in body weight, in mice with this dose, was tested for the H1N1 strains. Infection with the 10,000 TCID_50_ dose resulted in higher peak titers, across all three strains, than those noted with the 300 TCID_50_ dose (Supplementary Figures S5A and S7). The mutant strains seemed less pathogenic, as they resulted in lower body weight loss (rgH1N1.PA/I38T, maximum of 13.9% at 6 days post-infection and the rgH1N1.NA/H275Y, maximum of 17.0% at 7 days post-infection) than the rgH1N1.WT (maximum of 27.1% at 9 days post-infection). Furthermore, all mice infected with the mutant strains survived, whereas some mice infected with the WT strain died (or reached the weight of the humane endpoint [70% of the final body weight on the day of the infection]) (Supplementary Figure S8).

Next, mice infected with rgH1N1.WT, rgH1N1.PA/I38T, or rgH1N1.NA/H275Y (10,000 TCID_50_) were treated at 5 days post-infection. Body weight was observed for up to 10 days post-infection. Treatment with baloxavir acid demonstrated a significant inhibitory effect on loss of body weight, compared with vehicle (*p* < 0.05), on different days on 10 mg/kg and 30mg/kg doses. This effect was observed in rgH1N1.WT-infected mice on days 9 and 10 (10 mg/kg and 30 mg/kg doses), in rgH1N1.PA/I38T-infected mice on days 6 and 7 (30 mg/kg dose), and in rgH1N1.NA/H275Y-infected mice on days 6–9 (10 mg/kg dose) and days 6–10 (30 mg/kg dose). The inhibitory effect on loss of body weight was significantly greater with baloxavir acid than with oseltamivir phosphate in rgH1N1.NA/H275Y-infected mice on day 6 (10 mg/kg dose) and on days 6–8 (30 mg/kg dose) (Supplementary Figure S9).

## 4. Discussion

This is the first report investigating the in vivo antiviral effect of baloxavir acid at doses simulating human plasma concentrations in animal (mouse and hamster) models infected with H1N1, H1N1pdm09, and H3N2 virus subtypes with PA/I38T substitution. These mutant strains exhibited a similar level of in vivo viral replication as their respective WT strains in the non-lethal animal model used in this and previous studies [11]. Baloxavir acid resulted in a modest reduction in lung virus titers in animals infected with PA/I38T strains compared with those infected with the WT strains. This effect was similar to that observed with oseltamivir phosphate. Since very little weight loss and no lethality were observed in mice infected with PA/I38T strains, we were unable to establish a clear effect of baloxavir acid treatment in reducing clinical signs of infection in these mice.

We noted that the antiviral effect of baloxavir acid was numerically lower in mice infected with the PA/I38T strains compared with the WT and NA/H275Y strains. Baloxavir acid reduced the virus titers in mice infected with the WT strains, at doses as low as 1 mg/kg, but not in those infected with the PA/I38T strains.

It was demonstrated that the viral strains used in this study proliferate under the test condition. It is therefore noteworthy that, under these conditions, even with reduced efficacy against PA/I38T strains, the effect of baloxavir acid, at doses equivalent to clinical administration (10 mg/kg and 30 mg/kg) [27], was significantly greater than that seen with vehicle, as well as comparable to that of oseltamivir phosphate at clinically relevant doses (5 mg twice daily).

No viral rebound was observed in mice treated with baloxavir acid for up to 14 and 8 days post-infection with rgH1N1.PA/I38T and H1N1pdm09.PA/I38T, respectively. These findings suggest that a single dose of 30 mg/kg baloxavir acid was sufficient to reduce the virus titers in mice infected with PA/I38T strains at 6 dpi, leading to viral elimination.

We conducted a PK/PD analysis on PA/I38T infections in mice to strengthen the validity of the findings in the animal model and to facilitate translation into clinical situations. At doses simulating human plasma concentrations, baloxavir acid demonstrated a dose-dependent effect against PA/I38T infections, supporting its potential efficacy against PA/I38T mutant strains. The C_τ_ value was found to be a strong predictive PK parameter for antiviral activity of baloxavir acid against both rgH1N1.WT and rgH1N1.PA/I38T infections [11]. The C_τ_ value of baloxavir acid, which showed a similar in vivo antiviral effect against rgH1N1.PA/I38T infection as oseltamivir phosphate, was 31.6 ng/mL. We found that this plasma concentration was almost achieved in humans at 24 h in a non-Asian population and at 48–60 h in an Asian population, as determined in a previous study [27].

The pathogenicity of the PA/I38T strain was assessed by inoculating mice with a high infectious dose of the PA/I38T strain. The loss of body weight was observed at doses of 3000 TCID_50_ and higher. To compare the pathogenicity of the three strains, the mice were inoculated with 10,000 TCID_50_ of each strain. While the WT strain demonstrated body weight loss and lethality in infected mice, the loss in body weight and lethality was limited with the same dose of mutant viruses (NA/H275Y and PA/I38T), demonstrating lower pathogenicity. Even though baloxavir acid demonstrated a marginal inhibitory effect on weight loss in mice infected with the mutant strains, this effect may have been limited due to the low pathogenicity of the mutant viruses. Therefore, no clear effect of baloxavir acid on pathogenesis could be evaluated.

Previous experiments in mice, hamsters, and ferrets have shown that variants carrying the PA/I38T mutation demonstrate pathogenicity and replicative abilities that are similar to their respective WT strains, with a minor loss in fitness, and they have a lower transmission ability when mixed with the WT strains [17,18]. This relative fitness cost has been shown to be greater in H1N1pdm09 viruses than in H3N2 viruses. Using reverse genetic mutant strains, as opposed to clinical isolates, for a consistent genetic background, the PA/I38T mutation demonstrated a similar impact in this study. The growth of the mutant strain compared with the respective WT strains was not significantly different at the sub-lethal dose of 300 TCID_50_, confirming the replicative fitness and pathogenicity of the rgH1N1.PA/I38T mutant strain.

Our study has several limitations. First, owing to differences in the EC_50_ assay methodology, it is not possible to directly compare the EC_50_ of the mutant strains tested in this study with that of strains reported in other studies. The mutant isolates used in this study have shown an EC_50_-based fold change (FC) in baloxavir acid susceptibility comparable to those reported to date (27–57) [14]. However, a study that used clinical isolates revealed higher EC_50_-based FC values (65–155) [24] than our current results (57). Therefore, further investigation to determine whether baloxavir acid is effective against clinical isolates of different strains is recommended.

Second, in this study, we have only tested the PA/I38T strains. Surveillance data have identified other H3N2 and H1N1pdm09 strains with PA amino acid substitutions, such as E23K/G/R, A36V, A37T, I38M/F/L, E119D, and E199G, resulting from amino acid substitutions in the PA [14]. However, as these strains demonstrate a lower EC_50_-based FC than the PA/I38T strains, in susceptibility to baloxavir acid, we expect baloxavir acid to be substantially more effective against these mutant strains than the PA/I38T strain.

Third, our findings from this study are based on baloxavir acid treatment 5 days post-infection, followed by lung virus titer determination at 6 dpi. Additional experiments are needed to evaluate baloxavir acid efficacy earlier in the therapeutic regimen, as well as explore the prophylaxis effect when used pre-infection. In addition, we suggest that future experiments should be conducted to measure the virus spread in the lungs of infected mice, at different dpi, to further evaluate the drug efficacy.

Finally, the replication efficiency of influenza virus variants in the lungs can differ from drug-susceptible WT strains. We therefore propose that a 50% lethal dose should be determined in mice for each virus (a) to fully evaluate the antiviral efficacy of baloxavir acid in animal models, (b) to achieve comparable virus titers, and (c) to observe the clinical signs of infection caused by drug-resistant and drug-susceptible viruses.

In summary, to the best of our knowledge, this is the first study reporting the effects of baloxavir on infections caused by the PA/I38T mutant strain in mouse and hamster models. Our study showed that, upon clinically equivalent drug exposure in mice, baloxavir acid demonstrated minor, dose-dependent in vivo antiviral activity against PA/I38T mutant strains. Even though the degree of lung virus titer reduction with baloxavir acid was diminished in animal models infected with PA/I38T mutant strains, the observed antiviral effect was comparable to that of oseltamivir phosphate.

## Figures and Tables

**Table 1:** Drug susceptibility of WT and mutant strains of H1N1 and H3N2 viruses to baloxavir acid and oseltamivir acid.

Virus Subtype	Virus Strain	Baloxavir Acid			Oseltamivir Acid		
		EC_50_ (nmol/L)		FC	IC_50_ (nmol/L)		FC
		Mean	SD		Mean	SD	
H1N1 (rgA/WSN/33)	WT	0.42	0.12	N/A	0.80	0.09	N/A
	NA/H275Y	0.32	0.06	0.77	181.65	5.13	**226.12**
	PA/I38T	11.37	1.85	**27.24**	0.80	0.01	1.00
H1N1pdm09 (A/Osaka/129/2009)	WT	0.61	-	N/A	ND	ND	ND
	PA/I38T	15.7	-	**25.74**	ND	ND	ND
H3N2 (rgA/Victoria/3/75)	WT	1.13	0.51	N/A	0.13	0.02	N/A
	PA/I38T	63.80	3.40	**56.59**	0.16	0.01	1.21

The EC_50_ of baloxavir acid against H1N1pdm09 WT and PA/I38T strains was determined by a plaque reduction assay. The remaining data were taken from a previous study [14]. Fold change (FC) was calculated as relative EC_50_ or IC_50_ of each tested virus to that of the WT virus. Bold type, FC > 10; EC_50_, half-maximal effective concentration; IC_50_, half-maximal inhibitory concentration; N/A, not applicable; ND, no data; SD, standard deviation; WT, wild type.

## Data Availability

We will share the data upon reviewing the request. Requests of data sharing from researchers will be reviewed with regard to the validity and feasibility of the research intended.

## Supplementary Materials

The following supporting information can be downloaded at: https://www.mdpi.com/article/10.3390/v15051154/s1, Figure S1: Time course of virus titers in mice infected with H1N1 PA/I38T strain; Figure S2: Dose-dependent pharmacokinetics of baloxavir acid in non-infected mice and hamsters; Figure S3: Comparison of plasma concentrations of baloxavir acid in humans, mice and hamsters; Figure S4: Virus growth curves in mice infected with H1N1 PA/I38T strain; Figure S5: Viral growth curves in mice infected with influenza A at non-lethal doses; Figure S6: Body weight change in mice infected with different doses of H1N1 PA/I38T strain; Figure S7: Virus growth curves in mice infected with H1N1 strains; Figure S8: Body weight loss in virus-infected mice; Figure S9: Effect of baloxavir acid treatment on body weight change in mice infected with H1N1 WT, PA/I38T or H275Y strains; Table S1: List of primer sequences used for RT-PCR and sequencing; Table S2: Mean values of the parameters used in the PK/PD analysis of rgH1N1.PA/I38T-infected mice; Table S3: Analysis of pharmacokinetic parameters of baloxavir acid in the linear model.
