# Supplementary material for: In Vivo Antiviral Activity of Baloxavir against PA/I38T-Substituted Influenza A Viruses at Clinically Relevant Doses
Source: Viruses. 2023 May 11;15(5):1154. doi: 10.3390/v15051154 (PMC10222987; doi:10.3390/v15051154)
Supplement: Supplementary file 1 [file viruses-15-01154-s001.zip › viruses-2346086-supplementary.pdf]

Supplementary Material

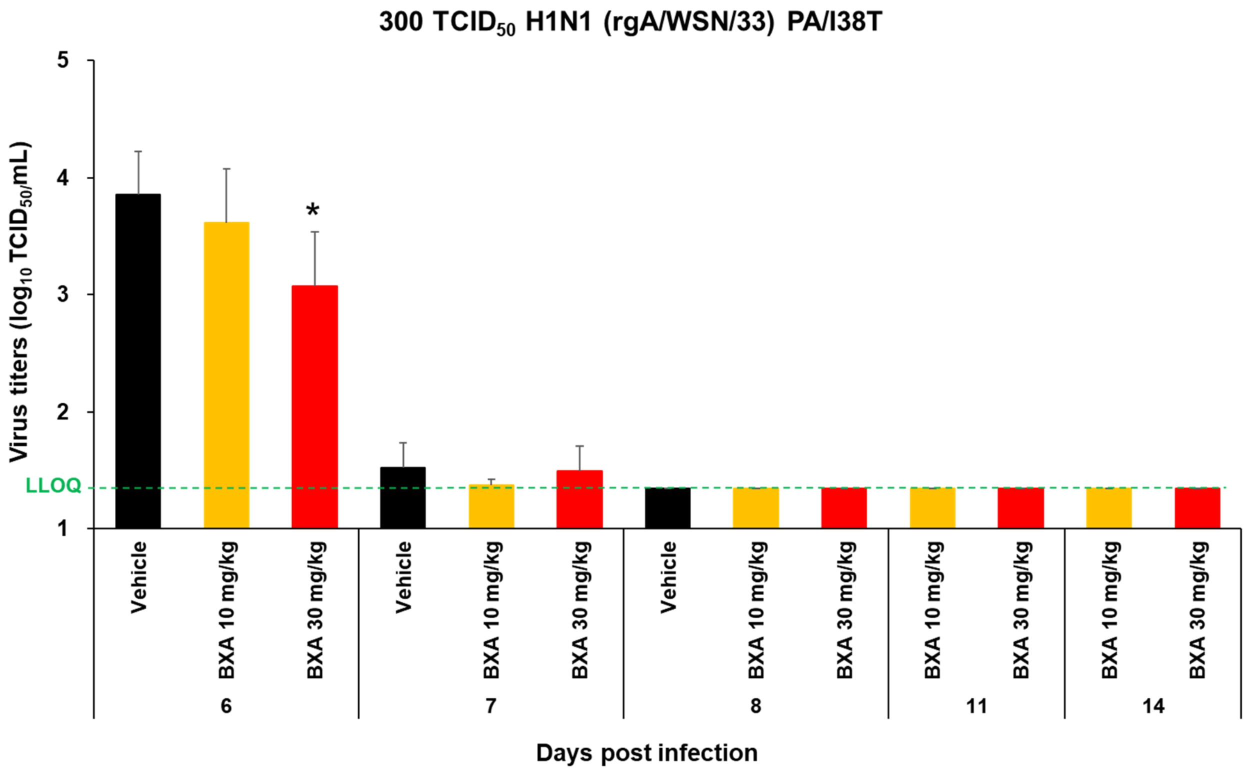

Supplementary Figure S1. Time course of virus titers in mice infected with H1N1 PA/I38T strain. Mice infected with 300 $TCID_{50}$ rgH1N1.PA/I38T were treated with baloxavir acid (10 or 30 mg/kg qd) or vehicle 5 days post infection. Lung virus titers were measured on Days 6, 7, 8, 11 and 14 post infection. The green dotted line shows the LLOQ. Each bar represents the mean ± standard deviation of 5 mice; **p* < 0.05 vs vehicle (Dunnett's test). BXA, baloxavir acid; $TCID_{50}$, median tissue culture infectious dose.

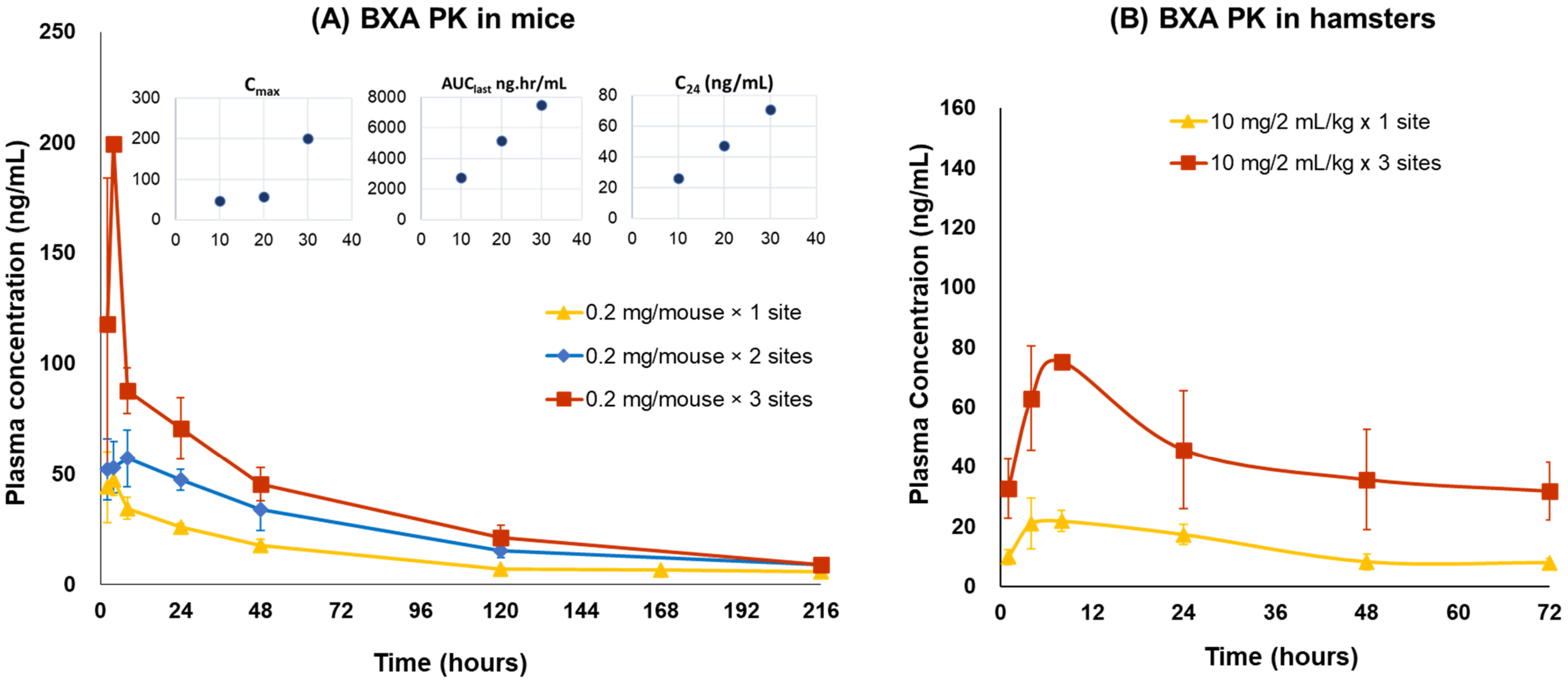

Supplementary Figure S2. Dose-dependent pharmacokinetics of baloxavir acid in non-infected mice and hamsters. (**A**) In mice, baloxavir acid suspension (1 mg/mL) was administered subcutaneously to one (≈10 mg/kg, yellow line, triangles), two (≈20 mg/kg, blue line, diamonds) or three (≈30 mg/kg, red line squares) sites on the back. Plasma concentrations were determined using blood taken from the inferior vena cava, heart or tail vein. (**B**) In hamsters, baloxavir acid suspension (5 mg/mL) was administered subcutaneous to one (10 mg/kg, yellow line, triangles) or three (30 mg/kg, red line squares) sites on the back. Plasma concentrations were determined using blood taken from the jugular vein. The

graphs (**A** and **B**) show the mean ± standard deviation of 3 mice and 3 hamsters. AUC_{last}, area under the curve up to the last measurable concentration; BXA, baloxavir acid; C_{24}, plasma concentration at 24 hours after the first dosing; C_{max}, maximum drug concentration; PK, pharmacokinetics.

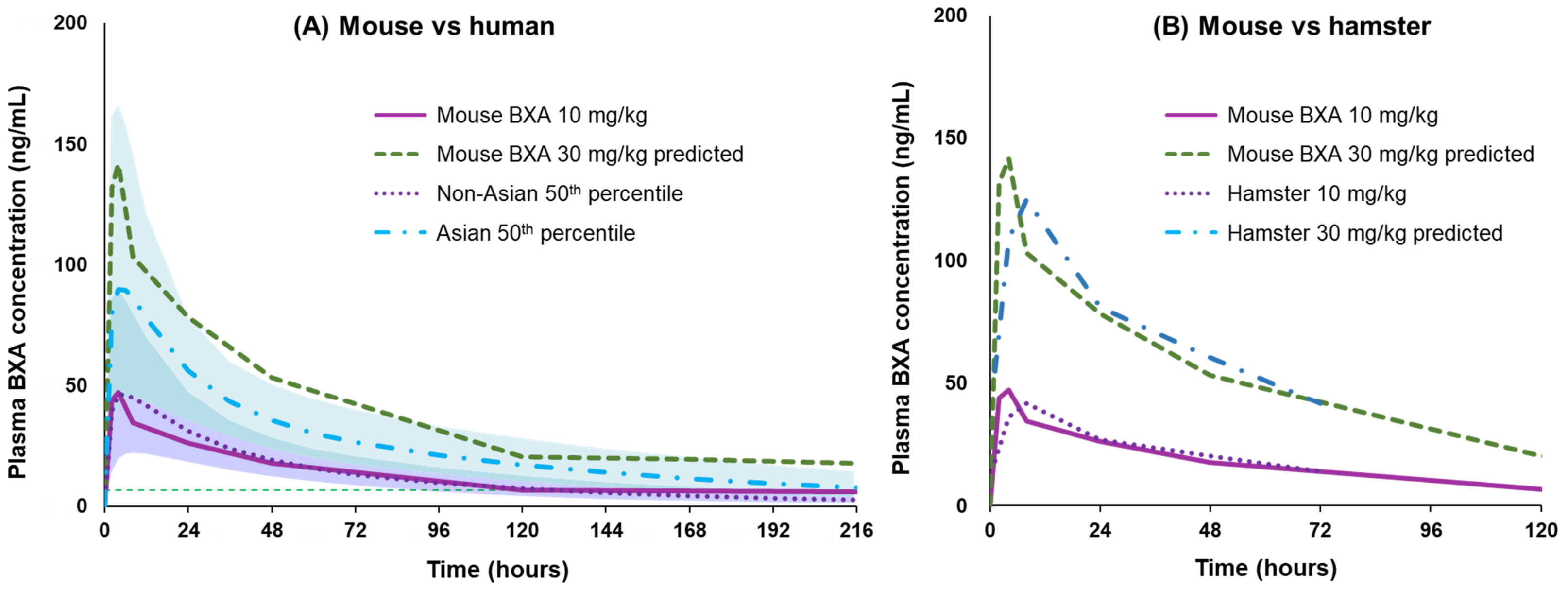

Supplementary Figure S3. Comparison of plasma concentrations of baloxavir acid in humans, mice and hamsters. Subcutaneous injections of baloxavir acid were administered at one site (10 mg/kg) on the back of mice or hamsters. Plasma concentrations of baloxavir acid for a 30 mg/kg dose in mice and hamsters were estimated from the measured plasma concentration data for the 10 mg/kg dose. Human pharmacokinetic data

were derived from a previous study [**26**]. The magenta solid line represents mouse PK at the 10 mg/kg dose and the green dashed line represents simulated mouse-predicted PK at the 30 mg/kg dose. (**A**) The dotted purple line represents the 50th percentile for the non-Asian population, the blue dash-dotted line represents the 50th percentile for the Asian population, the top and bottom of the shaded area represent the 90th and 10th percentiles of the human PK, respectively. (**B**) The dotted purple line represents the hamster PK at the 10 mg/kg dose, the blue dash-dotted line represents the simulated hamster PK at the 30 mg/kg dose. BXA, baloxavir acid.

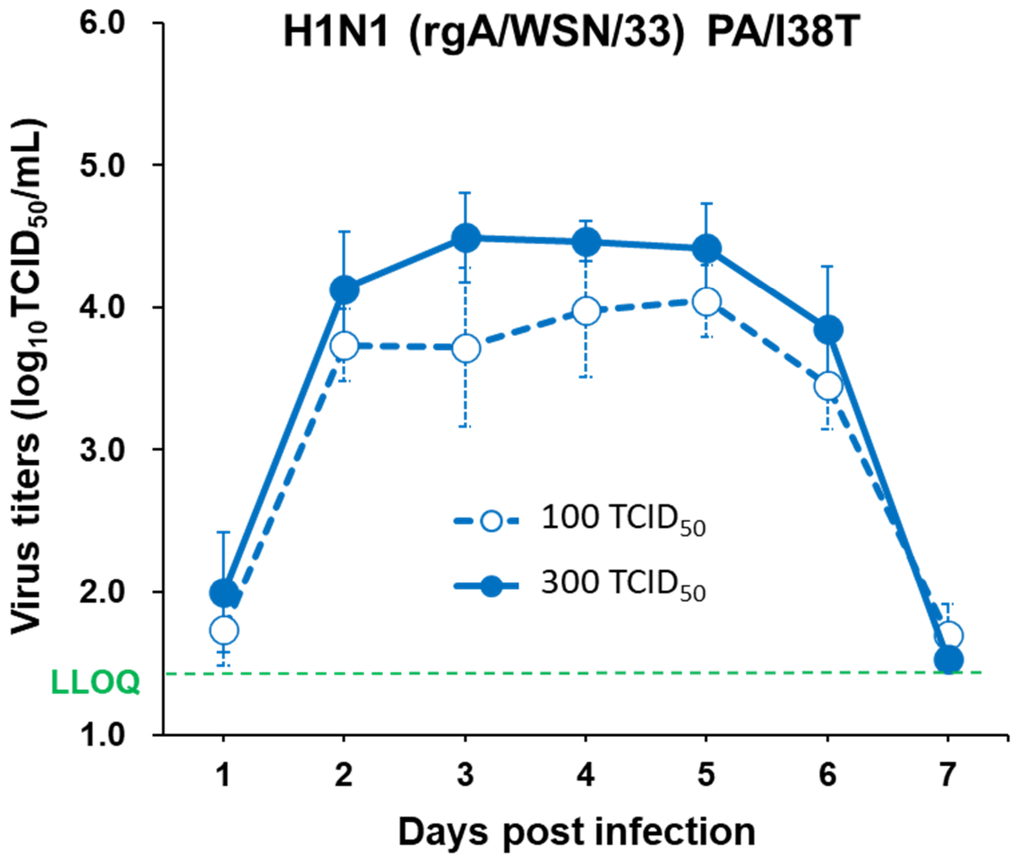

Supplementary Figure S4. Virus growth curves in mice infected with H1N1 PA/I38T strain. Mice were infected with rgH1N1.PA/I38T (100 and 300 $TCID_{50}$), and lung virus titers were measured 1–7 days post infection. The green dotted line shows the LLOQ. Data represent the mean ± standard deviation of 5 or 10 mice infected with 100 $TCID_{50}$ (open circles) or 300 $TCID_{50}$ (filled circles) rgH1N1.PA/I38T. LLOQ, lower limit of quantification; $TCID_{50}$, median tissue culture infectious dose.

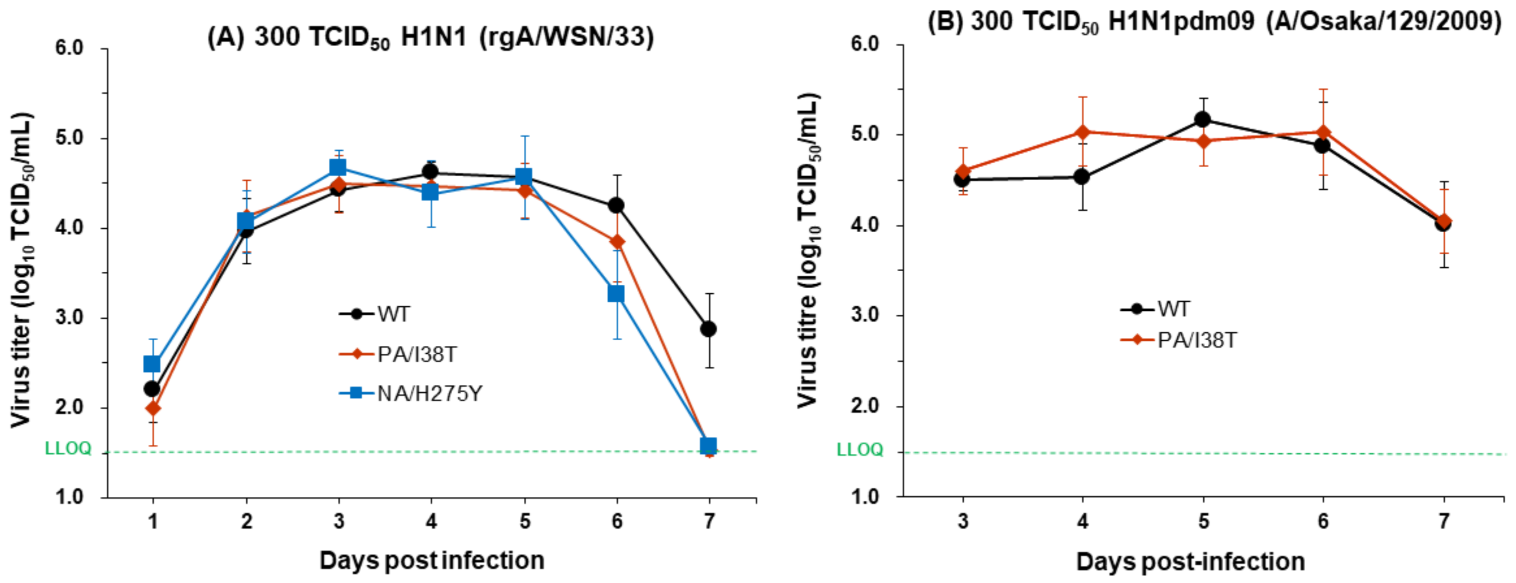

Supplementary Figure S5. Viral growth curves in mice infected with influenza A at non-lethal doses. (**A**) Mice were infected with 300 $TCID_{50}$ rgH1N1.WT (black line, circles), rgH1N1.PA/I38T (red line, diamonds) or rgH1N1.NA/H275Y (blue line, squares), and lung virus titers were measured on Days 1–7 post infection. (**B**) Mice were infected with 300 $TCID_{50}$ rgH1N1pdm09.WT (black line, circles) or rgH1N1pdm09.PA/I38T (red line, diamonds), and lung virus titers were measured on Days 3–7 post infection. Data represent the mean ± standard deviation of 5–10 mice. The green dotted line shows the LLOQ. LLOQ, lower limit of quantification; $TCID_{50}$, median tissue culture infectious dose; WT, wild type.

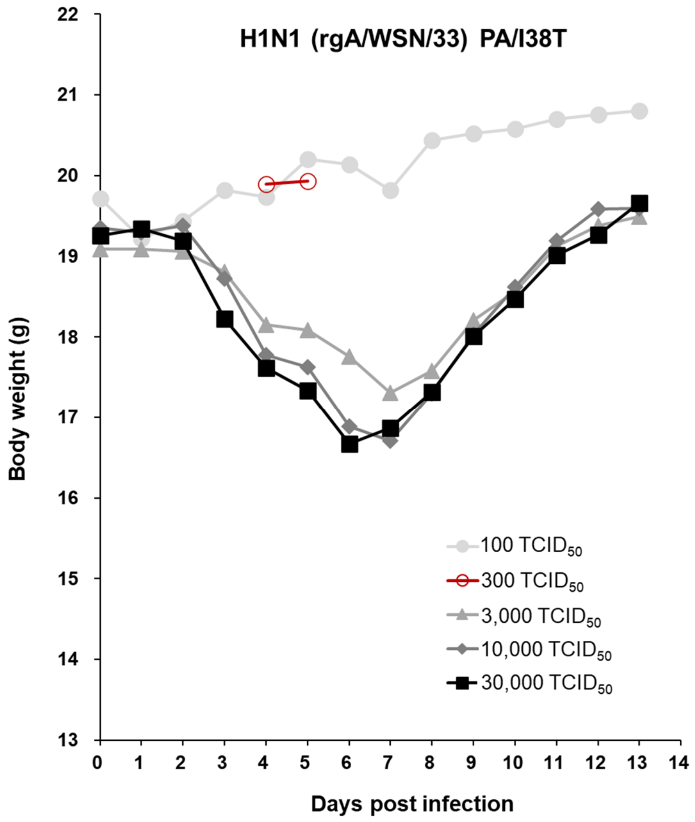

Supplementary Figure S6. Body weight change in mice infected with different doses of H1N1 PA/I38T strain. Mice were infected with 100, 300, 3,000, 10,000 and 30,000 $TCID_{50}$ rgH1N1.PA/I38T and the body weights were measured for 14 days (from Day 0 to Day 13). The filled circles indicate 100 $TCID_{50}$, the open circles indicate 300 $TCID_{50}$, the triangles indicate 3,000 $TCID_{50}$, the diamonds indicate 10,000 $TCID_{50}$, the squares indicate 30,000 $TCID_{50}$. $TCID_{50}$, median tissue culture infectious dose.

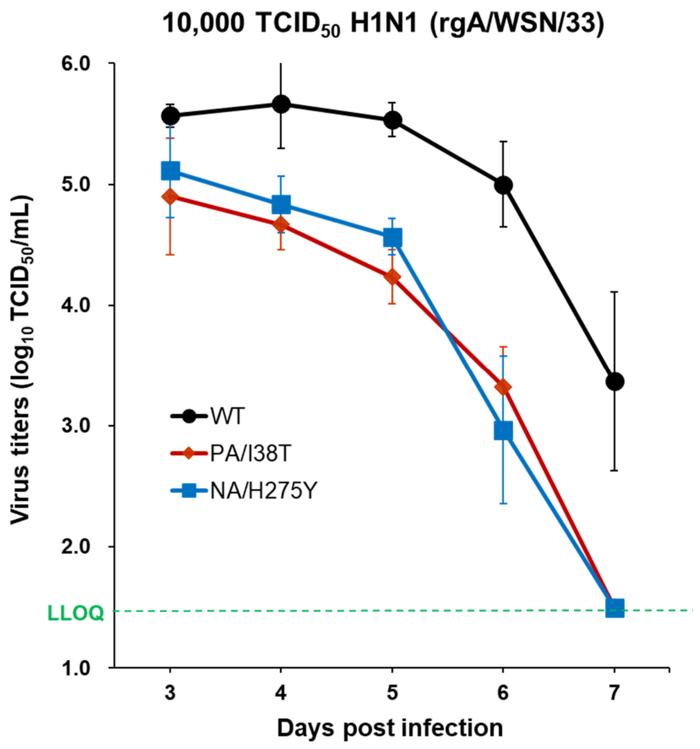

Supplementary Figure S7. Virus growth curves in mice infected with H1N1 strains. Mice were infected with 10,000 $TCID_{50}$ rgH1N1.WT (black line, circles), rgH1N1.PA/I38T (red line, diamonds) or rgH1N1.NA/H275Y (blue line, squares), and lung virus titers were measured on 3–7 days post infection. The green dotted line shows the LLOQ. Data represent the mean ± standard deviation of 5 mice. LLOQ, lower limit of quantification; $TCID_{50}$, median tissue culture infectious dose; WT, wild type.

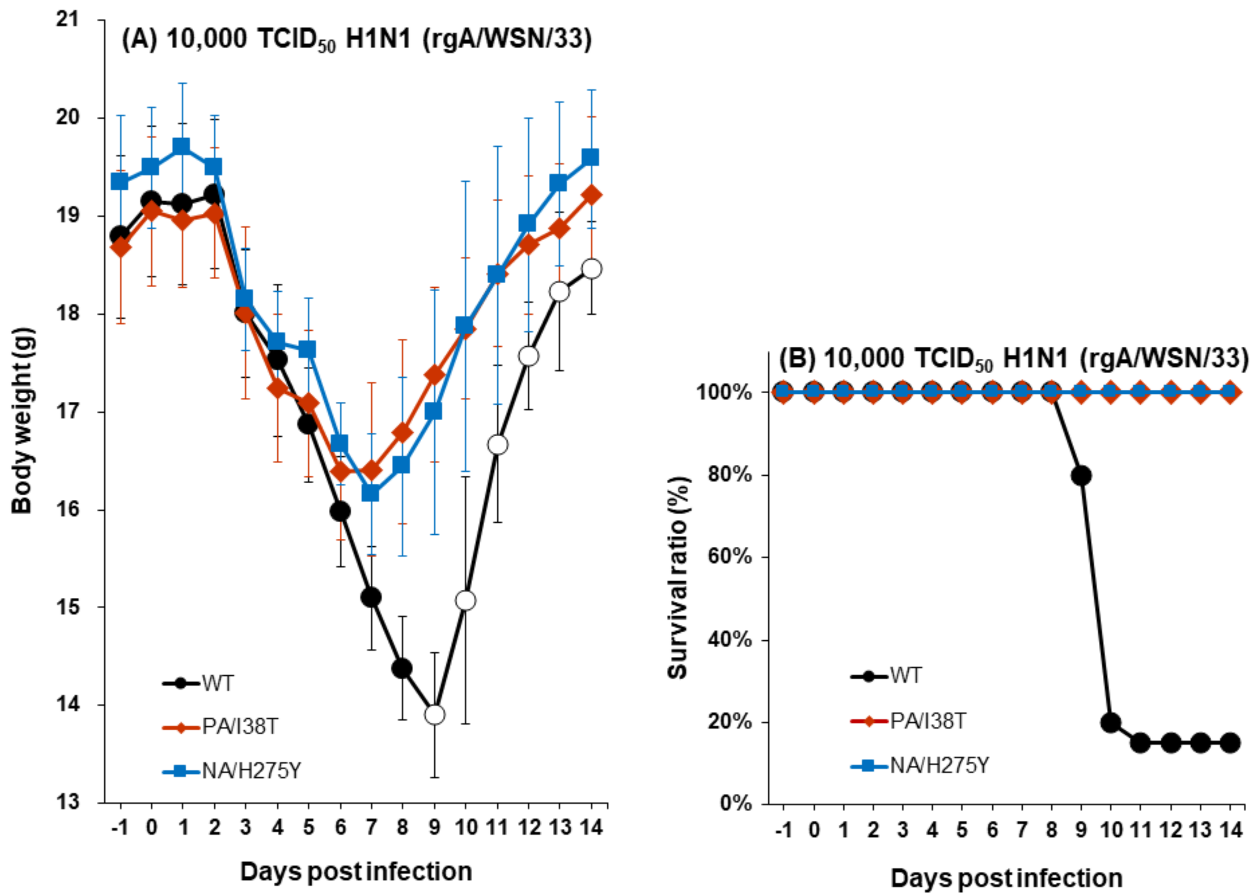

Supplementary Figure S8. Body weight loss in virus-infected mice. Mice infected with 10,000 $TCID_{50}$ rgH1N1.WT (black line, circles), rgH1N1.PA/I38T (red line, diamonds) or rgH1N1.NA/H275Y (blue line, squares) were examined daily for body weight and survival for 14 days

post infection. Open circles indicate that one or more mice died before observation. (**A**) Actual body weight over time in mice. Data represent the mean ± standard deviation of 10–20 mice. (**B**) Survival ratio of mice. $TCID_{50}$, median tissue culture infectious dose; WT, wild type.

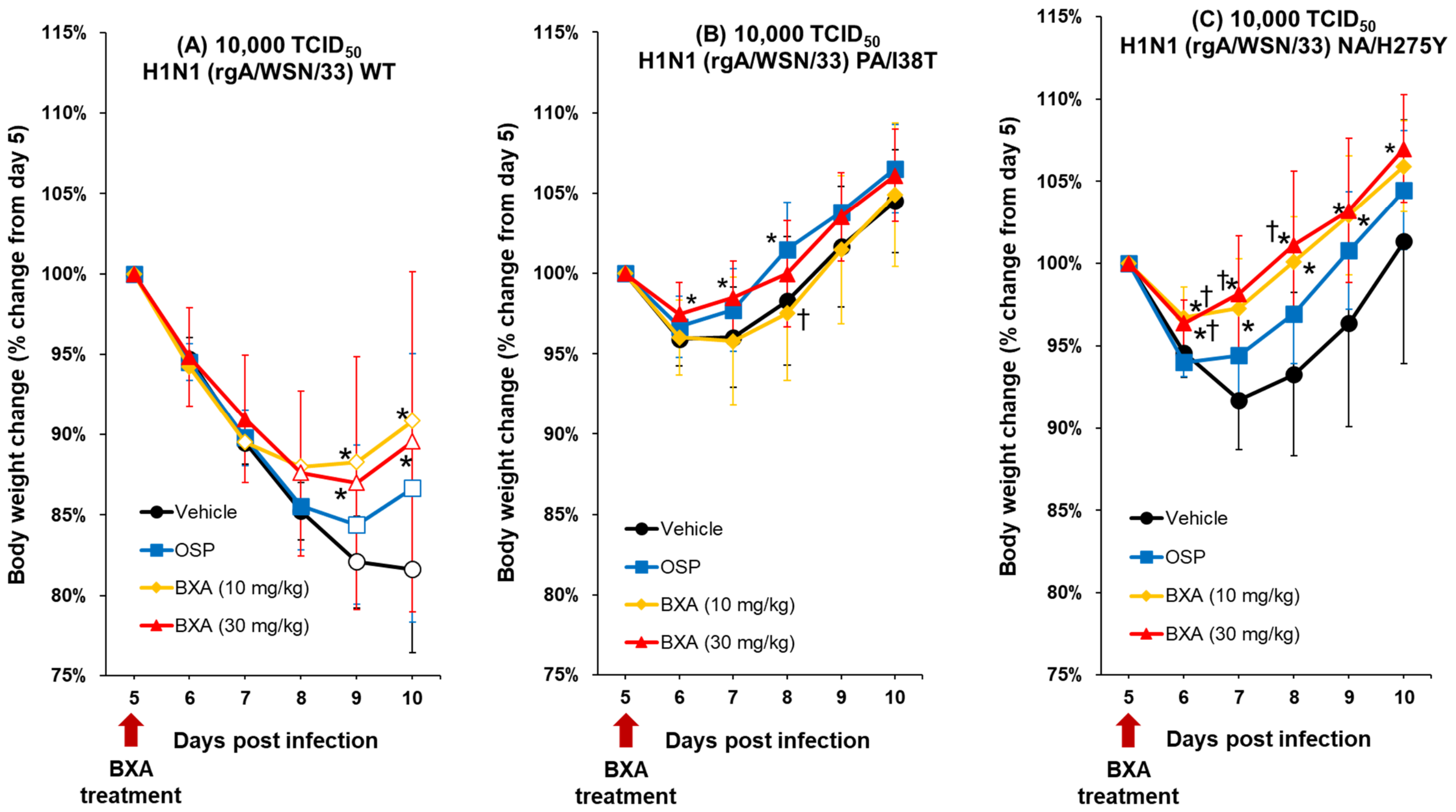

Supplementary Figure S9. Effect of baloxavir acid treatment on body weight change in mice infected with H1N1 WT, PA/I38T or H275Y strains. Mice infected with 10,000 $TCID_{50}$ (**A**) rgH1N1.WT, (**B**) rgH1N1.PA/I38T or (**C**) rgH1N1.NA/H275Y were treated with baloxavir acid

(10 or 30 mg/kg qd for 1 day), oseltamivir phosphate (5 mg/kg bid for 5 days) or vehicle (for 5 days) 5 days post infection. Body weight change was expressed as a percentage of baseline (i.e. the body weight on the first day of treatment, 5 days post infection). Data represent the mean ± standard deviation of 10–20 mice. *$p < 0.05$ vs vehicle; †$p < 0.05$ vs oseltamivir phosphate (Dunnett's test). The black lines with circles represent vehicle, the blue lines with squares represent OSP, the yellow lines with diamonds represent baloxavir acid (10 mg/kg), and the red lines with triangles represent BXA (30 mg/kg). Open symbols indicate that one or more mice died before observation. bid, twice daily; BXA, baloxavir acid; OSP, oseltamivir phosphate; qd, once daily; WT, wild type.

Supplementary Table S1. List of primer sequences used for RT-PCR and sequencing

	Gene	Primer name	Primer sequence (5' to 3')
**RT-PCR**	rgA/WSN/33 (H1N1)-PA	WSN-PAseg-1-M13F(1-21)	TGTAAAACGACGGCCAGTAGCGAAAGCAGGTACTGATTC
		WSN-PAseg-1-M13R(753-773)	CAGGAAACAGCTATGACCGACATTTGAGAAAGCTTGCCC
	A/Osaka/129/2009 (H1N1pdm09)-PA	WSN-PAseg-1-M13F(1-21)	TGTAAAACGACGGCCAGTAGCGAAAGCAGGTACTGATTC
		Osaka-PAseg-M13R(211-230)	CAGGAAACAGCTATGACCGCTTCAATAGTGCATTCGGGT
**Sequencing**	rgA/WSN/33 (H1N1)-PA and A/Osaka/129/2009 (H1N1pdm09)-PA	M13-F	TGTAAAACGACGGCCAGT
		M13-R	CAGGAAACAGCTATGACC

RT-PCR, reverse transcription polymerase chain reaction.

Supplementary Table S2. Mean values of the parameters used in the PK/PD analysis of rgH1N1.PA/I38T-infected mice

	Dose	C_τ	C_{24}	C_{max}	AUC_{0-24}	Virus titer	
	**mg/kg**	**ng/mL**	**ng/mL**	**ng/mL**	**h*ng/mL**	**Log_{10} $TCID_{50}$/mL**	
						**mean**	**SD**
**BXA qid**	0.125	3.20	11.5	18.7	267	3.94	0.52
	0.25	6.39	23.0	37.3	535	3.88	0.34
	0.5	17.5	37.4	66.1	961	3.73	0.26
	1	39.8	66.1	124	1814	3.45	0.17
	2	84.9	135	222	3626	3.30	0.55
	4	175	274	417	7251	2.13	0.59
**BXA bid**	0.5	10.9	14.1	43.7	539	3.86	0.27
	1	17.0	19.6	82.4	1003	3.62	0.39
	2	33.5	38.1	141	2006	3.71	0.44
	4	66.6	75.2	258	4014	3.07	0.43
	8	127	160	414	6385	2.56	0.65
	16	247	330	726	11128	1.63	0.29
**BXA qd**	2	4.61	4.61	122	1288	3.95	0.34
	4	8.56	8.56	224	2587	3.79	0.35
	8	33.3	33.3	334	4053	3.21	0.44
	16	82.7	82.7	554	6987	2.40	0.68
	32	160	160	682	8855	1.88	0.38
	64	315	315	938	12591	1.56	0.12

The mean values of the parameters for the PK/PD analysis to which the linear model was applied. AUC_{0-24}, area under the curve from 0 to 24 hours; bid, twice daily; BXA, baloxavir acid; C_τ, plasma concentration at the end of the dosing interval after the first dosing; C_{24}, plasma concentration at 24 hours after the first dosing; C_{max}, maximum plasma concentration; PK/PD, pharmacokinetic/pharmacodynamic; qd, once daily; qid, four times daily; SD, standard deviation; $TCID_{50}$, median tissue culture infectious dose.

Supplementary Table S3. Analysis of pharmacokinetic parameters of baloxavir acid in the linear model ($y = E_0 + \beta x$)

PK parameter	Model parameter	Estimate	Standard error	95% CI		*p*-value	R^2
**AUC_{0-24} (ng・h/mL)**	E_0	8.208	0.330	7.555	8.861	< 0.0001	0.633
	β	-0.652	0.042	-0.735	-0.570	< 0.0001	
**C_{max} (ng/mL)**	E_0	6.495	0.235	6.029	6.960	< 0.0001	0.606
	β	-0.652	0.044	-0.739	-0.564	< 0.0001	
**C_{24} (ng/mL)**	E_0	5.350	0.154	5.046	5.653	< 0.0001	0.627
	β	-0.574	0.037	-0.647	-0.500	< 0.0001	
**C_τ (ng/mL)**	E_0	5.043	0.122	4.801	5.285	< 0.0001	0.672
	β	-0.541	0.032	-0.604	-0.479	< 0.0001	

AUC_{0-24}, area under the curve from 0 to 24 hours; β, regression coefficient; CI, confidence interval; C_τ, plasma concentration at the end of the dosing interval after the first dosing; C_{24}, plasma concentration at 24 hours after the first dosing; C_{max}, maximum plasma concentration; E_0, baseline effect; PK, pharmacokinetic; R^2, coefficient of determination.
